# Antibacterial and Antifungal Properties of *Ocotea indecora* Essential Oil and Its Nanoemulsion

**DOI:** 10.3390/ph18121909

**Published:** 2025-12-18

**Authors:** Francisco Paiva Machado, Julia C. Scaffo, Leonardo A. Pinto, Renata F. A. Pereira, Sorele Fiaux, Luiz Antonio M. Keller, Eduardo Ricci-Júnior, Ana Paula dos Santos Matos, Fabio Aguiar-Alves, Caio P. Fernandes, Jorge A. D. Duarte, Leandro Rocha

**Affiliations:** 1 Laboratório de Tecnologia de Produtos Naturais (LTPN), Universidade Federal Fluminense (UFF), Niterói 24241-000, Rio de Janeiro, Brazil; fmachado@id.uff.br (F.P.M.); leandromr@id.uff.br (L.R.); 2 Laboratório de Epidemiologia Molecular e Biotecnologia (LEMB), Laboratório Universitário Rodolfo Albino, Niterói 24241-000, Rio de Janeiro, Brazilfaalves@gmail.com (F.A.-A.); 3 Laboratório de Tecnologia Microbiana, Departamento de Tecnologia Farmacêutica, Faculdade de Farmácia, Universidade Federal Fluminense (UFF), Niterói 24241-000, Rio de Janeiro, Brazil; 4 Departamento de Zootecnia e Desenvolvimento Agrosustentável, Faculdade de Medicina Veterinária, Universidade Federal Fluminense (UFF), Niterói 24230-321, Rio de Janeiro, Brazil; 5 Laboratório de Desenvolvimento Galênico (LADEG), Departamento de Fármacos e Medicamentos, Faculdade de Farmácia, Universidade Federal do Rio de Janeiro (UFRJ), Rio de Janeiro 21941-599, Rio de Janeiro, Brazil; 6 Institute of Drug Technology-Farmanguinhos, Oswaldo Cruz Foundation (Fiocruz), Rio de Janeiro 21040-900, Rio de Janeiro, Brazil; 7 Department of Pharmaceutical Sciences, Lloyd L. Gregory School of Pharmacy, Palm Beach Atlantic University, West Palm Beach, FL 33401, USA; 8 Laboratory of Phytopharmaceutical Nanobiotechnology, Department of Biological and Health Sciences, Federal University of Amapá, Macapá 68902-280, Amapá, Brazil; 9 CIAB Research Group, School of Agricultural, Livestock and Environmental Sciences (ECAPMA), National Open and Distance University (UNAD), CEAD Ocana, Ocana 54670, Colombia

**Keywords:** *Thielaviopsis ethacetica*, antibacterial, antifungal, Lauraceae, *Staphylococcus aureus*

## Abstract

**Background:** Antimicrobial resistance and fungal contamination remain major threats to public health and agriculture, emphasizing the need for innovative alternatives. Plant-derived products are a promising alternative, and nanoformulations may further enhance their activity. **Objective:** This study investigated the antimicrobial potential of *Ocotea indecora* essential oil and its nanoemulsion. **Methods/Results:** The essential oil chemical characterization by GC-MS revealed sesquirosefuran (91.61%) as the main constituent. A factorial design guided the selection of an optimized nanoemulsion, which exhibited spherical nanometric droplets (79 nm and 0.029 PdI) with long-term stability. The essential oil inhibited the growth of Gram-positive and Gram-negative strains at 1 to 2 mg/mL, while the nanoemulsion enhanced bactericidal activity against *Staphylococcus aureus*. In contrast, antifungal assays revealed a more pronounced effect, with the nanoemulsion lowering the minimum inhibitory concentrations (625 µg/mL) against *Thielaviopsis ethacetica*, thereby enhancing the inhibitory activity of the essential oil (2.5 mg/mL). Morphological alterations, including thinner hyphae and impaired sporulation, were also detected, suggesting a reduction in fungal virulence. **Conclusions:** In summary, *O. indecora* essential oil shows promising antimicrobial potential, and nanoemulsification proved particularly effective in potentiating fungistatic activity while offering limited enhancement of bactericidal effects. The results support the potential of *O. indecora* derivatives as natural candidates for the development of novel antimicrobial strategies.

## 1. Introduction

Bacteria and fungi represent major threats to human and agricultural health due to their role as infectious agents, toxin producers, and food contaminants [1]. Healthcare-associated infections are a major concern in hospitals, especially among newborns and patients in intensive care units, due to their increased vulnerability and exposure to invasive procedures [2]. The ESKAPE group, comprising *Enterococcus faecium*, *Staphylococcus aureus*, *Klebsiella pneumoniae*, *Acinetobacter baumannii*, *Pseudomonas aeruginosa*, and *Enterobacter* spp., exhibits high levels of antimicrobial resistance and complicates treatment strategies [3]. Similarly, fungi contribute to global health and economic burdens through infections, toxin production, and post-harvest losses in food systems, reinforcing the urgent need for innovative approaches to prevention and control [1,4].

The evolutionary dynamics of bacteria and fungi, shaped by natural selection, have driven the emergence of resistance to antimicrobial and phytosanitary agents [5,6]. In bacteria, factors such as the improper use of antibiotics, self-medication, inadequate treatment duration, and delays in diagnostic methods promote the widespread use of broad-spectrum antibiotics, accelerating resistance [6]. Similarly, fungi also develop resistance, worsened by the continued use of the same chemical agents for decades in agriculture [5]. One of the most prominent phytopathogens affecting tropical crops such as pineapple, coconut, banana, sugarcane, and oil palm is the *Thielaviopsis ethacetica* Went., the anamorph phase of *Ceratocystis paradoxa* (Dade) C. Moreau [7,8]. This scenario highlights the urgent need for innovative antimicrobial and phytosanitary products that are safe and environmentally friendly, aiming to address the growing resistance observed in clinically relevant bacterial strains as well as in fungal pathogens responsible for significant agricultural losses worldwide [4,9,10].

All these challenges underscore the urgent need for novel strategies to control microbial contamination and infections without compromising patient safety, food security, or environmental sustainability [11]. While conventional approaches often rely on synthetic additives and pesticides, their adverse effects on human health, non-target organisms, and ecosystems have raised growing concerns [12,13]. In this context, natural products represent promising alternatives due to their rich diversity of bioactive compounds and, suggestively, lower environmental impact [14]. Among them, essential oils stand out as complex mixtures of secondary metabolites with antimicrobial potential, whose mechanisms of action include disruption of cell and mitochondrial membranes, interference with ion transport, modulation of membrane receptors and proteins [15,16,17].

Essential oils can be integrated with advanced biotechnological approaches to enhance their functional activities. Among these, oil-in-water nanoemulsions have emerged as promising delivery systems, characterized by an internal oil phase dispersed within an external aqueous phase in the form of droplets, typically with diameters ranging from 20 to 200 nm [18]. These nanostructured systems exhibit notable long-term physical stability and significantly enhance the solubility and dispersion of hydrophobic bioactive compounds [19]. This improved solubility facilitates more effective interactions between the encapsulated plant derivatives and target microorganisms, thereby enhancing antimicrobial efficacy [20].

*Ocotea indecora* (Shott) Mez (Lauraceae) is a neotropical native and endemic plant from Brazil and is popularly known in the north of Rio de Janeiro as “canela-sassafrás”. Previous studies have described sesquirosefuran as the main sesquiterpenoid in the chemical profile of the essential oil from the leaves obtained in the north of Rio de Janeiro [21,22]. The biological potential of the essential oil from leaves has been described with acaricidal effects in *Rhipicephalus* (*Boophilus*) *microplus* [21] and insecticidal properties against *Aedes aegypti*, *Drosophila suzukii*, and *Dysdercus peruvianus* [22,23,24]. Regarding microbiological properties, until the present moment, there is only one study that describes the fungistatic effects of the essential oil in *Aspergillus* species [25]. Therefore, this study provides a comprehensive evaluation of the antibacterial and antifungal potential of *O. indecora* essential oil and its oil-in-water nanoemulsion, expanding current knowledge on its antimicrobial spectrum. By exploring its efficacy against clinically relevant bacteria and agriculturally significant fungi, we seek to establish *O. indecora* as a promising natural source of bioactive compounds and to assess whether nanoemulsion-based delivery enhances its stability and antimicrobial potential.

## 2. Results

### 2.1. Essential Oil Characterization

**Figure 1:**
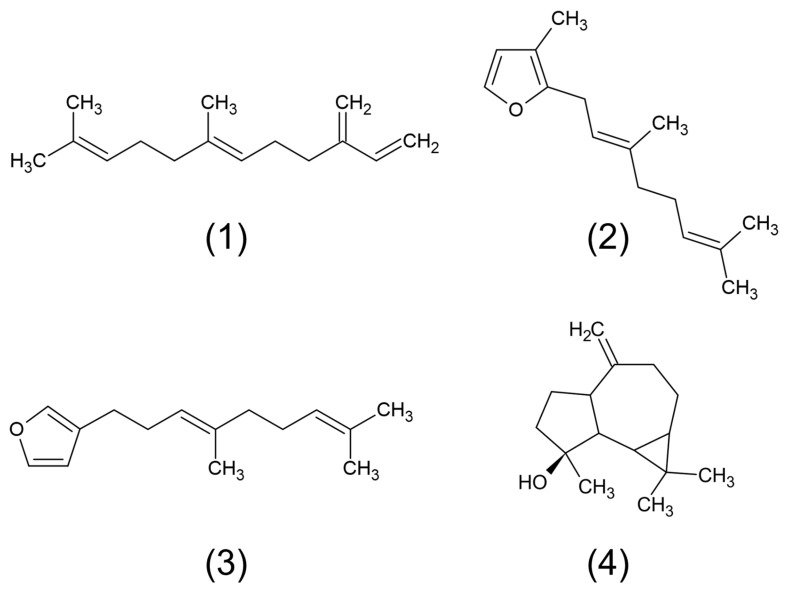
*Ocotea indecora* essential oil components: (**1**) β-farnesene; (**2**) sesquirosefuran, (**3**) dendrolasin, and (**4**) spathulenol.

The essential oil (yielding 0.64%, *w*/*w*) was characterized by GC-MS, enabling the tentative identification of four substances (Figure 1), accounting for 96.29% of the *O. indecora* essential oil (Table 1). The sesquiterpene sesquirosefuran was the major component (91.61%), followed by β-farnesene (2.95%). The chromatogram can be seen in Supplementary Materials Figure S1.

### 2.2. Experimental Design

**Figure 2:**
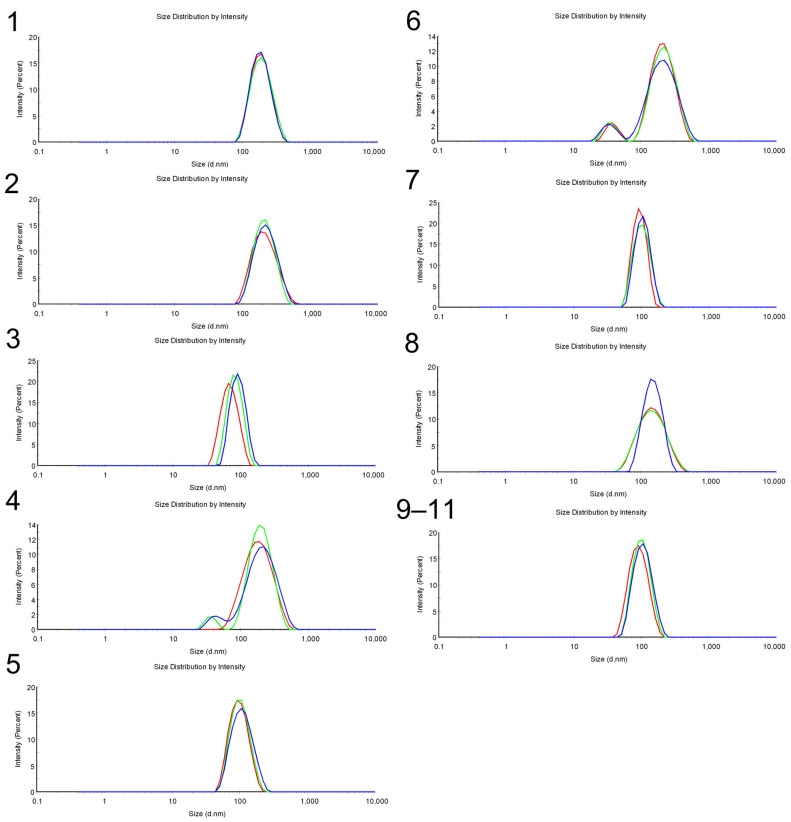
Droplet size distribution by intensity of the nanoformulations 1 to 11. The blue, red, and green lines represent the three independent replicates (*n* = 3) of the DLS analysis for each formulation (1 to 8), demonstrating reproducibility. Formulations 9 to 11 correspond to the replicated center point of the DOE, and the displayed size distribution by intensity graph is the mean value of these three replicates, being red for formulation 9, green for formulation 10, and blue for formulation 11.

The matrix and responses of the 2^3^ experimental designs are presented in Table 2. All formulations exhibited droplet sizes ranging from 72 to 198.5 nm and polydispersity index (PdI) values ranging from 0.029 to 0.287 after preparation. Figure 2 displays the droplet size distribution by intensity for all prepared formulations. Formulations 1–3, 5, 7–11 exhibited monomodal behavior, whereas formulations 4 and 6 displayed bimodal distributions.

**Figure 3:**
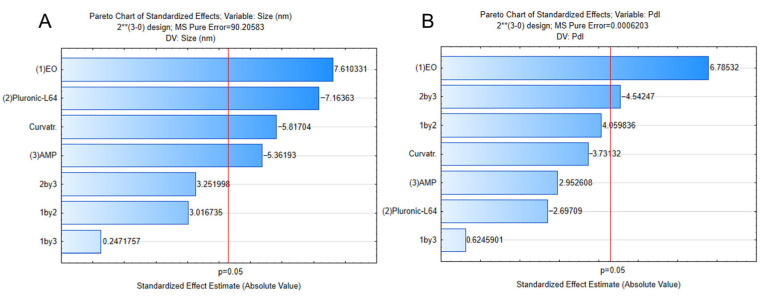
Pareto charts of standardized effects for the factorial 2^3^ design, showing the relative influence of oil concentration, Pluronic L64 concentration, and sonication amplitude, as well as their interactions, on (**A**) droplet size and (**B**) polydispersity index (PdI) of *O. indecora* nanoemulsions. The vertical line indicates the threshold of statistical significance (*p* < 0.05).

The factorial 2^3^ design with curvature adjustment, analyzed against pure error, revealed that all three main factors (oil concentration, Pluronic L64 concentration, and sonication amplitude) significantly affected droplet size (*p* < 0.05), with the model showing excellent fit (R^2^ = 0.98; Adj. R^2^ = 0.94). For PdI, oil concentration (*p* = 0.021) and its interaction with amplitude (*p* = 0.045) were significant (Figure 3). In both cases, the lack-of-fit test was not significant, confirming that the adjusted models adequately captured the experimental data. These findings indicate that all parameters influenced droplet size. Whereas PdI is primarily driven by oil concentration, with additional contributions from factor interactions (BC), reflecting the non-linear dynamics of droplet generation in nanoemulsified systems. The detailed ANOVA results are provided in Table S1 of the Supplementary Materials.

**Figure 4:**
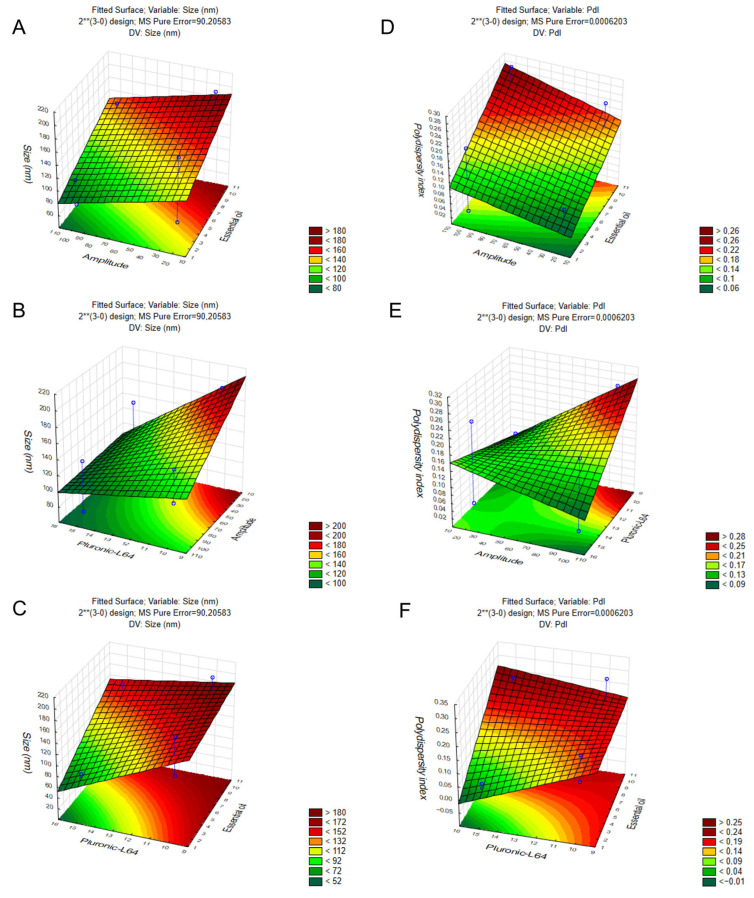
Three-dimensional response surface plots for the effects of formulation and variables on *O. indecora* nanoemulsions. Panels (**A**–**C**) show the effects of (**A**) amplitude x oil concentration, (**B**) Pluronic L64 x amplitude, and (**C**) Pluronic L64 x oil concentration on droplet size. Panels (**D**–**F**) show the corresponding effects of (**D**) amplitude x oil concentration, (**E**) Pluronic L64 x amplitude, and (**F**) Pluronic L64 x oil concentration on the polydispersity index (PdI). In each plot, the third factor was fixed at its central level (oil concentration = 6%, Pluronic L64 = 12.5%, amplitude = 60).

The response surface plots revealed distinct trends for droplet size and polydispersity index (PdI) (Figure 4). For droplet size, the smallest values were generally observed in regions with lower oil concentration, higher Pluronic L64 levels, and increased sonication amplitude, indicating that both formulation and processing parameters act synergistically to promote efficient droplet disruption (Figure 4A–C). For PdI, lower values were associated with reduced oil concentration, while interactions between surfactant concentration and amplitude further contributed to narrowing the droplet size distribution (Figure 4D–F). Taken together, these results indicate that the most favorable conditions for generating a nanoemulsion with smaller droplets and lower PdI are achieved at the lowest oil concentration tested (2%), the highest surfactant concentration (15%), and the highest sonication amplitude (100).

**Figure 5:**
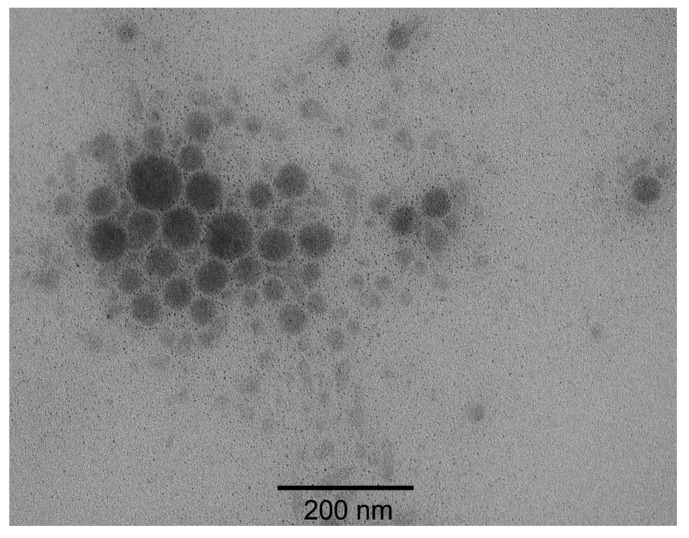
Transmission electron microscopy (TEM) of *O. indecora* essential oil nanoemulsion (Ne-OiOE) droplets.

Based on the formulation results, the most promising formulation of *O. indecora* nanoemulsion (Ne-OiOE, 2% EO, 15% Pluronic L-64, amplitude 100) was selected and subsequently subjected to transmission electron microscopy (TEM) analysis. At 89,000× magnification, the TEM revealed spherical droplets with nanometric dimensions, predominantly below 120 nm, in agreement with the DLS data. The images also showed a relatively uniform morphology, corroborating the narrow size distribution indicated by the low PdI values (0.029 ± 0.021) (Figure 5).

### 2.3. Nanoemulsion Stability

The Ne-OiOE was stable at room temperature (25 °C ± 2), presenting transparent-bluish characteristics, maintaining homogeneous average droplet size (76.56–104 nm), and PdI (0.015–0.058) values after 90 days of preparation (Table 3). The Ne-OiOE stored under refrigeration (8 °C ± 2) showed a milky aspect with increased viscosity during storage. This fact corroborates the thermal plasticity of the surfactant polymer Pluronic L-64. In this way, the nanoemulsion stored under refrigeration was kept at room temperature (25 °C) before the DLS analysis, to return to the initial macroscopic characteristics (transparent blue liquid) with stable parameters, showing acceptable stability after 90 days of preparation, showing a droplet size increase (78.36–130 nm, *p* < 0.0001) and higher PdI (0.020–0.210, *p* < 0.0001) increase from 90 days of storage. Lastly, the Ne-OiOE in the climatic chamber (40 °C ± 2 °C) showed instability throughout the 90 storage days, showing creaming after 30 days, and gradually increasing the size of the droplets and PdI over time (*p* < 0.0001). Figure S2 shows the Ne-OiOE droplet size distribution by intensity when stored at (A) 25 °C, (B) 8 °C, and (C) 42 °C, over the 90 days after preparation.

### 2.4. Antibacterial Assay

The minimal inhibitory concentrations (MICs) of the essential oil and nanoemulsion were determined by microdilution assay and are presented in Table 4. The essential oil and nanoemulsion of *O. indecora* did not show inhibition in Gram-negative strains (*E. coli*, *E. cloacae*, *P. aeruginosa*, and *K. pneumoniae*) at the concentration tested (2 mg/mL). The Gram-positive strains were inhibited up to 2048 µg/mL, except for *S. aureus* (ATCC 25923) methicillin-sensitive (MSSA), which showed inhibition at 1024 µg/mL for both essential oil and nanoemulsion (Ne-OiOE); however, the nanoemulsion showed a lower bactericidal effect (1024 µg/mL) when compared to the essential oil (2048 µg/mL). Vancomycin exhibited inhibition of 1 µg/mL to the Gram-positive strains, while ciprofloxacin showed <3.9 µg/mL for the Gram-negative strains. The blank nanoemulsion (without essential oil) and negative control (DMSO 1%) did not exhibit bacterial inhibition.

### 2.5. Antifungal Assays

#### 2.5.1. Fungistatic Activity

**Figure 6:**
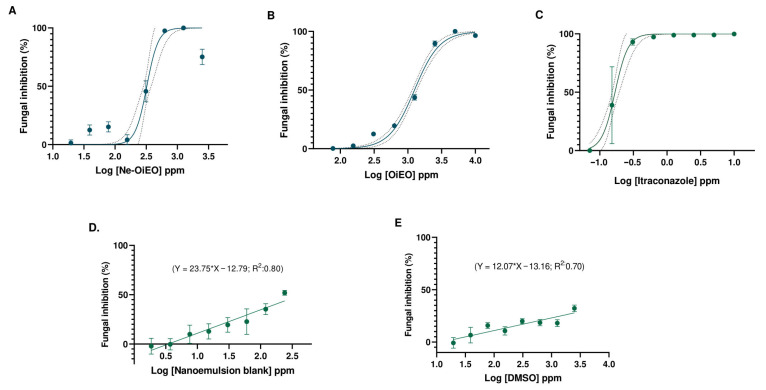
Dose–response curves against *Th. ethacetica*. The curves for treatments *Ocotea indecora* essential oil nanoemulsion (Ne-OiEO, (**A**), *Ocotea indecora* essential oil (OiEO, (**B**), and itraconazole (Itra, (**C**) were fitted using non-linear regression with normalized response. The normalization of fungal inhibition percentages was based on responses from the same treatment. Dotted lines around the sigmoidal curve indicate confidence/prediction bands at the 95% confidence level. Data for the nanoemulsion blank, C-Ne (**D**), and Tween 80, C-EO (**E**), were fitted with linear regression. Each data point represents the mean of three determinations, and the error bars show the standard deviation.

The quantitative microdilution technique was used to assess the antifungal activity of *Ocotea indecora* essential oil (OiEO) and the nanoemulsion (Ne-OiEO) at various doses. Fungal growth was seen in all micro wells of the fungal blank (FB) and the negative controls, Tween 80 (C-EO) and nanoemulsion (C-Ne, without essential oil), at 90 h of incubation for *Th. ethacetica*, except those containing OiEO, Ne-OiEO, and itraconazole (Figure S3). The precise effect of the treatments at various concentrations on *Th. ethacetica* was evaluated by fitting a dose–response curve of vegetative growth inhibition of the pathogen (Figure 6). Under the same experimental settings, the fungal inhibition (%) took into account the absolute absorbance of the treatments and the absolute absorbance of the fungal control. Treatment absorbance differences on separate plates were detected for all treatments, particularly the nanoemulsion (Ne-OiOE) (Figure S3).

The fungistatic potential of the Ne-OiEO, OiEO, and itraconazole therapies was assessed using a previously reported qualitative and quantitative criteria. Both methodologies demonstrate that the Ne-OiEO has a fungistatic effect on *Th. ethacetica* at doses of 625 µg/mL, whereas OiEO only exhibited this effect at 2500 µg/mL on *Th. ethacetica* (Table 5). The treatments’ dose–response curves show coefficients of determination ranging from 0.70 to 0.96 and root mean square error (RMSE) ranging from 19.14 to 5.6, indicating that the nonlinear model fits the experimental data (Tables S2–S4). This model demonstrated that the nanoemulsion may elicit 50% inhibition (IC_50_) on *Th. ethacetica* at concentrations thrice and fivefold lower than when the essential oil (Table 5). However, when resazurin was applied to the microplates and aliquots of the treatments were reinoculated at fungistatic quantities on BDA nutrition plates, it was discovered that none of the treatments demonstrated fungicidal effect on the target phytopathogens (Figure S4).

#### 2.5.2. Fungal Micromorphology

The current study employed the poisoned medium technique to examine the influence of nutritional media polluted with treatments on growth and development of *Th. ethacetica* fungal structures. This anamorphic fungus produces aleurioconidia as well as two endoconidia known as secondary conidia and primary conidia. *Th. ethacetica* generated thick-walled, dark brown, oblong aleurioconidia (aC) with an average dimension of 12 × 6 μm when cultured on BDA medium at 25 °C for 72 h. The breadth of the smooth, hyaline vegetative hyphae averaged 3.87 μm. Secondary conidia were classified as aseptate, light brown, ovoid, with a typical size of 7 × 4 μm. In addition, lengthy chains of cylindrical hyaline primary conidia (4.3 × 1.0 μm) were found.

**Figure 7:**
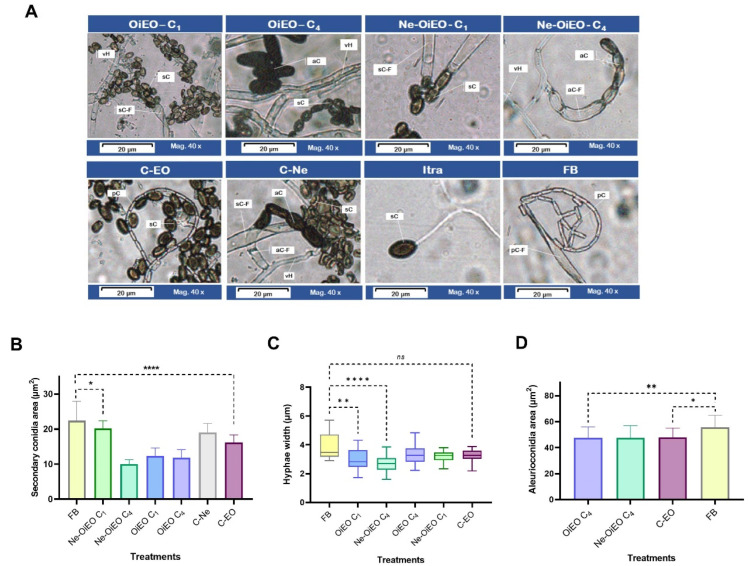
Morphological characterization of fungal structures of *Thielaviopsis ethacetica* exposed to plant and chemical treatments by absorption. Ne-OiEO C1 and C4: *Ocotea indecora* essential oil nanoemulsion at 2500 µg/mL and 325 µg/mL; OiEO C1 and C4: *Ocotea indecora* essential oil at 10,000 µg/mL and 1250 µg/mL; C-Ne: nanoemulsion blank; C-EO: Tween 80; Itra: itraconazole; FB: fungal blank. (**A**) Compiled microphotograph of the effect of *Th. ethacetica* structures such as hyphae (vH), aleurioconidia (aC), secondary conidia (sC), primary conidia (pC), phialidic vial-shaped conidiophore (secondary conidia-producing) (sC-F), lateral aleuroconidia-producing apex phialid (f-aC), apex of primary conidia-producing phialidic conidiophore (pC-F). (**B**) Comparison of the approximate area of *Th. ethacetica* secondary conidia. Significant differences: * 0.0186; **** <0.0001 according to Dunnett’s multiple comparisons test (*p* < 0.05). (**C**) Comparison of *Th. ethacetica* hyphal thickness; ns: not significant; significant differences ** 0.0037, **** <0.0001 based on Dunn’s multiple comparisons test. (**D**) Comparison of *Th. ethacetica* aleurioconidia area; * 0.0129, ** 0.008 based on Dunnett’s multiple comparisons test (*p* < 0.05).

Also, itraconazole and *O. indecora* essential oil (OiEO) affected the production of these fungal structures (Figure 7A). OiEO at 10,000 µg/mL disrupted the development of aleurioconidia and primary conidia, but at 1250 µg/mL, it only inhibited primary conidia sporulation (pC). Similarly, compared to nanoemulsion (Ne-OiEO) at 325 µg/mL, Ne-OiEO at 2500 µg/mL inhibited the formation of primary conidia and slowed the sporulation of aleurioconidia from short lateral phialides (aC-F). On the itraconazole-containing slide, secondary spores with narrow vegetative tubes were observed, which prevented the formation of the reported decreased vegetative mycelium. Significant changes in the median values of the secondary conidia dimension of *Th. ethacetica* were seen in all treatments to which *Th. ethacetica* was subjected according to analysis of variance (ANOVA) (*p* < 0.05) (Figure 7B, Chart S1). Furthermore, given the non-normal distribution of hyphal width measurements, the Kruskal–Wallis test revealed significant differences in the treatments used by *Th. ethacetica* during their growth (*p* < 0.05) (Chart S2). According to Dunn’s multiple comparisons test, Ne-OiEO (325 µg/mL) and OiEO (10,000 µg/mL) induced thinner hyphae compared to the fungal blank (Figure 7B). Although Ne-OiEO and OiEO at the lowest concentrations examined (C4) did not affect the production of aleurioconidia, Dunn’s multiple comparisons test revealed that they did alter the size of these conidia (Figure 7D, Chart S3).

## 3. Discussion

The essential oil from *O. indecora* leaves has been previously reported in the literature. Recent studies with *O. indecora* collected in the coastal sandbank environment of Rio de Janeiro, as the actual study, corroborate the presence of sesquirosefuran as the major compound (81.41% to 87.00%) of the essential oil obtained from its leaves, characterized by GC-MS and NMR [21,22,26,27]. However, when collected at São Paulo Atlantic Forest, the chemical profile exhibited bicyclogermacrene (29.79%), valerianol (15.12%), spathulenol (11.16%), and β-pinene (11.41%) [28]. Although it should be noted that since authentic standards were not available for the GC-MS analysis in the present study, compound assignments were made based on mass spectral similarity and retention index agreement with reference libraries, and should therefore be interpreted as tentative [29,30].

The metabolic variation between plant specimens is well documented, with different biotic and abiotic factors potentially influencing the qualitative and quantitative composition of secondary metabolites [31]. These combined factors contribute to a dynamic metabolic plasticity that reflects the ecological adaptation of plants, while simultaneously creating challenges for ensuring the standardization and reproducibility of plant-derived products [32]. In this context, the essential oil extracted from the leaves of *O. indecora*, obtained from the Restinga de Jurubatiba National Park in Rio de Janeiro, is suggestively consistent, as evidenced by the findings of the present study and those reported in the existing literature [22,23,24,25,26,27].

The practical application of essential oils is still challenging due to their inherent physical and chemical properties (e.g., lipophilicity, volatility). This poses a significant obstacle to the technical development of pharmaceutical and agricultural products [19,33]. Consequently, nanoemulsification has been a concept often applied to essential oils over the years [34]. This is because it enables their application as delivery systems. In addition, the utilization of nanoemulsified systems has been demonstrated to enhance biological activity due to increased bioavailability, permeation, and absorption [19].

For a colloidal system to be considered a nanoemulsion, the formulation must present an average droplet size between 20 and 200 nm, with a polydispersity index below 0.3 to be classified as a monodisperse system [35]. It is noteworthy that the range of results obtained from the nanoformulations through the experimental design showed desirable collective nanometer parameters (Droplet size: 72 to 198.5 nm; polydispersity index: 0.045 to 0.287), suggesting the attainment of nanoemulsified systems. In addition, the formulations exhibited desirable macroscopic characteristics, such as a bluish reflection, and those with a lower essential oil content had a translucent appearance [36].

The factorial design results are consistent with previous reports, which demonstrated that lower oil fractions combined with higher levels of non-ionic surfactants reduce interfacial tension and improve interfacial coverage, leading to smaller droplet sizes and narrower distributions [37]. The influence of sonication amplitude also agrees with ultrasonic emulsification theory, in which higher energy enhances droplet disruption and decreases average size [38]. Collectively, these findings are aligned with the literature and reinforce that the most favorable formulations are obtained with reduced oil concentration, elevated surfactant levels, and optimized amplitude, which together promote smaller droplets and acceptable polydispersity values [39].

The stability study of Ne-OiEO showed that, after 90 days of preparation, the nanoemulsions, when stored at room temperature (25 °C) and under refrigeration (8 °C), remained stable and did not exhibit signs of destabilization. In contrast, when stored at 42 °C, the elevated temperature may promote partial dehydration of the hydrophilic polyethylene oxide (PEO) blocks from Pluronic-L64 at the droplet interface, reducing the efficiency of steric stabilization [18]. Under these conditions, the increase in Brownian motion and the decrease in the viscosity of the continuous phase not only intensified droplet collisions but also accelerated creaming, as described by Stokes’ law [40]. Furthermore, higher temperatures favor Ostwald ripening, in which molecules diffuse from smaller to larger droplets, leading to the growth of droplet size and enhancing gravitational separation [41]. Altogether, these processes corroborate the intrinsic thermodynamic instability of nanoemulsions, while highlighting the suitability of Pluronic L64 as a surfactant for stabilizing *O. indecora* essential oil at lower storage temperatures.

The *Ocotea indecora* essential oil (OiEO), obtained from leaves and its nanoemulsion (Ne-OiEO), demonstrated inhibitory and bactericidal effects against Gram-positive strains at concentrations of 1 to 2 mg/mL. Although there are no reports of the antibacterial properties of the *O. indecora* essential oil in the literature, other studies have reported a similar inhibition range of action in bacterial strains when using essential oil from *Ocotea* species [42,43,44,45]. In this study, nanoemulsification did not improve the inhibition of bacterial growth but enhanced the bactericidal activity against *S. aureus* ATCC 25923. This finding suggests that the nanoemulsion facilitated the dispersion and bioavailability of lipophilic constituents without shifting the inhibitory threshold during the 24 h incubation period, possibly due to a delayed release profile typical of nanostructured systems [20].

One of the main outcomes of the present study was the improvement of antifungal activity. The fungistatic effect of Ne-OiEO (MIC = 625 µg/mL) was observed at a substantially lower concentration than the essential oil (MIC = 2500 µg/mL), confirming that nanoemulsification enhances fungal inhibition. Itraconazole, used as the reference drug, presented a MIC of 0.31 µg/mL, corresponding to an approximately 2016-fold difference when compared with the nanoemulsion. Similar results were described for *Myristica fragrans*, *Bunium persicum*, and *Zanthoxylum alatum* nanoformulations, which increased antifungal and anti-aflatoxin B1 activities against *A. flavus* [9]. Nevertheless, this enhanced effect is not always consistent in the literature. For instance, Cruz et al. (2018) reported that *Lippia gracilis* nanoemulsion exhibited higher MIC (600 µg/mL) values against *Thielaviopsis paradoxa,* the etiological agent of coconut tree stem bleeding, than the essential oil (230 to 260 µg/mL) [46]. Such contrasting evidence reinforces that the impact of nanoemulsification on antimicrobial efficacy is context-dependent, varying with oil composition, microorganism type, inhibition methodology, and droplet release behavior [47,48].

The biological activity of a phytocomplex is often primarily associated with its major constituent (e.g., sesquirosefuran); the potential contribution of minor components (e.g., spathulenol, dendrolasin, and β-farnesene) cannot be excluded [49]. Spathulenol-rich essential oils have been repeatedly reported to exhibit relevant antibacterial and antifungal effects against a variety of microorganisms, supporting their role as a bioactive sesquiterpene [50,51]. Thus, the presence of other constituents in the essential oil may exert additive or synergistic effects, contributing to the inhibitory response observed against the microorganisms evaluated in the present study.

The morphological features of *Th. ethacetica* identified in this study align with those described by Borges et al. (2019), Mbenoun et al. (2014), and Nascimento et al. (2020), who examined isolates of the pathogen in pineapple and cocoa in Cameroon, and sugarcane and oil palm in northern Brazil, respectively [7,8,52]. The study of conidia formation, which is responsible for pathogen spread, survival, and pathogenicity, is particularly important for aiding in the monitoring and integrated management of diseases in the agricultural field [7,8,52]. Although the fungistatic character of *O. indecora* essential oil, the present study demonstrates that when the plant derivative is absorbed by *Th. ethacetica*, it disrupts the pathogen’s mycelial growth, affecting nutrient absorption and resulting in hyphae and the formation of propagules. Also, OiEO influenced *Th. ethacetica* sporulation processes. At the highest concentration tested, these plant derivatives reduced the size of secondary conidia responsible for disease transmission and hindered the production of resistant aleurioconidia and primary conidia. The suppression of aleurioconidia formation suggests that applying essential oil may impair the pathogen’s survival and spread in the field, as these cells enable the fungus to survive harsh environmental conditions for up to 16 months in the soil [53]. Overall, this study highlights the potential of *O. indecora* essential oil and its nanoemulsion as promising natural agents for the development of antimicrobial strategies.

## 4. Materials and Methods

### 4.1. Plant Material

The research with *O. indecora* was registered at SisGen (A491A56) and SisBio/ICMBio (13659-21). The leaves of *O. indecora* were collected in the Restinga de Jurubatiba National Park (site “22°12.683′ S”, “41°35.283′ O”), Carapebus, Rio de Janeiro, Brazil. The plant was identified, and a specimen voucher was herborized and deposited in the University of the State of Rio de Janeiro (UERJ) herbarium (São Gonçalo, Rio de Janeiro, Brazil) under code RFFP: 16.873.

### 4.2. Essential Oil Extraction

The extraction of the essential oil was realized based on the methodology described by Pinto et al. (2023) [25]. The *O. indecora* leaves (581 g) were separated from the stems, crushed in 3 L of distilled water in a blender (SPL-052, Spolu-benesse, Itajobi, SP, Brazil), added to a 5 L round-bottom flask, and submitted to hydrodistillation for 3 h in a Clevenger-type apparatus to obtain the essential oil. Then, the oil was dehydrated with anhydrous sodium sulphate (Na_2_SO_4_, ≥ 99%, Sigma-Aldrich, St. Louis, MO, USA) and stored at −20 °C in a screw-top borosilicate amber glass vial with polypropylene caps.

### 4.3. Essential Oil Characterization

The chemical characterization of the essential oil was performed as described by Machado et al. (2024) [27] using a GC-MS QP2010 (Shimadzu, Kyoto, Japan) gas chromatograph equipped with a mass spectrometer to identify the essential oil components, and a GC-2014 (Shimadzu, Kyoto, Japan) gas chromatograph equipped with a flame ionization detector (FID) to determine the relative abundance. One microliter of the oil, dissolved in dichloromethane (1000 ppm, ≥99.9%, Sigma-Aldrich, St. Louis, MO, USA), was injected at 260 °C injector temperature. The carrier gas was helium at a 1 mL/min flow rate with split injection (1:20 ratio). The oven temperature was initially 60 °C and then increased to 290 °C at 3 °C/min. The chromatographic column was an RTX5 for MS (Restek Corporation, Bellefonte, PA, USA, 0.25 mm ID, 30 m in length, 0.25 μm). The mass spectrometry conditions were 70 eV electron ionization and 1 scan/s scan rate. Also, the FID temperature was 290 °C. The arithmetic index (AI) was calculated by interpolating the retention times of a mixture of aliphatic hydrocarbons (C7-C40) analyzed under the same chromatographic conditions from the sample. The identification of the substances was accomplished by comparing their AI and mass spectra with those reported in the literature [54,55]. The compound’s MS fragmentation pattern was also compared with NIST mass spectrum libraries. The relative abundance of the chemical constituents was determined by flame ionization gas chromatography (GC-FID) under the same conditions as GC-MS. Analysis and percentages of these compounds were obtained by the FID peak area normalization method.

### 4.4. Nanoemulsion Preparation and Characterization

The formulations were prepared based on Betzler et al. (2019) using 2 to 10% of *O. indecora* essential oil as the oil phase and 10 to 15% of Pluronic L-64^®^ (Sigma-Aldrich, St. Louis, MO, USA) as the aqueous phase [56]. Initially, the oil phase was dripped into the aqueous phases and homogenized in a Vortex (KASVI^®^, São José dos Pinhais, PR, Brazil model K40-1010) for 1 min, then nanoscaled in an Ultrasonic Processor (UP100H, Hielscher^®^, Teltow, BB, Germany) in cycle 1 with multiple amplitudes (20 to 100) for 1 min. All samples were prepared in an ice bath to dissipate heat.

The formulations were diluted in 1:20 with distilled water, and characterized at room temperature (25 °C) by the Dynamic Light Scattering (DLS) technique in a Nanosizer Nano^®^ S90 (Malvern, UK) to evaluate the parameters mean droplet size and polydispersity index (PdI). All analyses were performed in triplicate.

### 4.5. Experimental Design 2^3^

A factorial design was performed according Betzler et al. (2019) using the Statistica 12 software using as independent variables: amplitude (20–100), amount of essential oil (2–10%, *w*/*w*), and Pluronic-L64 (10–15%, *w*/*w*) to evaluate the influence on the dependent variables average droplet size (nm), and polydispersity index (PdI) to determine the best preparation conditions for obtain the essential oil nanoemulsion of *O. indecora* (Table S5) [56]. Model adequacy was verified by curvature check, and the final analysis considered the adjusted model. The most promising nanoemulsion (Ne-OiEO) was also prepared to observe the micromorphological aspects of the droplets using transmission electron microscopy (TEM, Morgagni 268.FEI, Hillsboro, OR, USA). Initially, the nanoemulsion was diluted in deionized water (1:1 proportion). Then, 5 µL was added to a copper grid with a formvar coating, dried for 1 h in a desiccator, and submitted to analysis.

### 4.6. Nanoemulsion Stability

The selected nanoemulsion (Ne-OiEO) was prepared in triplicate, stored in 5 mL screw-top amber borosilicate glass vials with black polypropylene plastic caps, and submitted to stability analysis at room temperature (25 °C), under refrigeration (8 °C), and in a climatic chamber (42 °C). The DLS analyses evaluated the parameters average droplet size (nm) and PdI at 0, 7, 15, 30, 60, and 90 days after preparation. The macroscopical characteristics (color, phase separation, cremation, sedimentation, and precipitation) were also observed.

### 4.7. Bacterial and Fungal Strains

The bacterial strains were from the Laboratory of Molecular Epidemiology and Biotechnology (LEMB) at Fluminense Federal University (Niterói, Rio de Janeiro, Brazil). Bacterial strains *Staphylococcus aureus* ATCC 25923 and USA 300, *Staphylococcus epidermidis* ATCC 122282, *Escherichia coli* ATCC 25922, *Enterobacter cloacae* ATCC 13047, *Pseudomonas aeruginosa* ATCC 27853, and *Klebsiella pneumoniae* ATCC 13883 were stored in Brain Heart Infusion (BHI) broth with 10% glycerol at −80 °C. *Thielaviopsis ethacetica* E-748 was provided from the Mycological Collection of the Instituto Capixaba de Pesquisa, Assistência Técnica e Extensão Rural INCAPER (Espirito Santo, Brazil), and the access was registered at SISGEN (AAA459F).

### 4.8. Antibacterial Evaluation

The Minimum Bactericidal Concentration (MIC) was performed in triplicate using the standard broth microdilution method under laboratory conditions (CLSI M07-A10). Strains were streaked in Muller-Hinton Agar (MHA) plates, and after 24 h, colonies were transferred to a 0.85% NaCl solution and adjusted to a 0.5 McFarland standard scale. The *O. indecora* essential oil was diluted in 1% DMSO and applied in 96-well plates with Muller-Hinton Broth (MHB), followed by a serial dilution ranging in concentrations from 2 mg/mL to 0.015 mg/mL. The blank nanoemulsion (without essential oil) and DMSO control were also evaluated. Vancomycin and ciprofloxacin were used as positive controls for the Gram-positive and the Gram-negative bacteria, respectively. Plates were incubated overnight at 37 °C for 24 h, and 20 µL of resazurin dye was added to reveal the results. After MIC incubation, the Minimum Bacterial Concentration (MBC) was evaluated. Aliquots of 10 µL from the wells were applied to Petri plates with MHB, divided into fields corresponding to each concentration of the microdilution, including a positive control. The endpoint is defined as the last quadrant without bacterial growth after incubation for 24 h at 37 °C, indicating 99.9% elimination of bacteria.

### 4.9. Antifungal Evaluation

The antifungal activity against *Th. ethacetica* was evaluated by microdilution based on Souza et al. (2020) [57]. The tests were conducted in sterile 96-well microtiter plates filled with 100 µL of Sabouraud Dextrose Broth (SDB) nutrient medium at a double concentration and 100 µL of each treatment in duplicate. Serial concentrations obtained were 2500 to 19.53 µg/mL for *Ocotea indecora* essential oil nanoemulsion (Ne-OiEO) and blank nanoemulsion (C-Ne); 10,000 to 78 µg/mL for *O. indecora* essential oil (OiEO) dissolved in Tween 80; 242 to 1.8 µg/mL for negative control Tween 80 (C-EO), and 10 to 0.07 µg/mL of itraconazole (positive inhibition control). Each treated well received 10 µL of spore suspension (1 × 10^6^ conidia/mL). SDB containing fungal inoculum was used in the fungal blank wells, while SDB medium and sterile distilled water were used in the sterility control wells. Two additional plates with treatments without inoculum were prepared to assess the background absorbance of each treatment. The plates are incubated at 25 °C for 90 h in the dark. Figure S5 shows a schematic of the treatment distribution in the microplates.

The growth-inhibition ratio was determined by measuring the absorbance at 630 nm of the micro-well in each plate using an automated plate reader (Thermo Plate Reader, Pittsburgh, PA, USA). After 90 h of incubation, the apparent absorbance values (relative to fungal growth) in each well with treatments at different concentrations were used to calculate the percent inhibition using Equation (1).

(1) $$Fungal\;inhibition\left(\%\right)=100-\frac{100\times \left(A_{630}\;of\;treated\;well-Average\;A_{630}\;of\;treatment\;background\;absorption\right)}{\left(A_{630}\;of\;growth\;well-Average\;A_{630}\;of\;medium\;backgraund\;absorpton\right)}$$

The dose–response curve for each treatment was constructed considering the normalized percentage inhibition (response) and the logarithm (base 10) of each treatment, expressed in µg/mL (*w*/*v*). The dose–response curve of treatments containing oil and itraconazole was determined using a nonlinear regression model based on Equation (2). Meanwhile, the solvent control data followed the linear regression model (Equation (3)). The sigmoid curve has a variable slope (Hill slope—As) and a normalized response comparing the effect of each treatment on *Th. ethacetica*. The parameters *As* and IC_50_ were calculated using the Levenberg–Marquardt algorithm for linear least squares minimization and GraphPad Prism v.9.0 (GraphPad Software, San Diego, CA, USA).

(2) $$Y=\frac{\left(100\right)}{1+10^{\left[Log\left({IC}_{50}-X\right)*\left(As\right)\right]}}$$

(3) Y=mx+b

The minimum inhibitory concentration (MIC) is the lowest concentration of the treatment at which no apparent fungal growth occurs in quadruplicate, with 80% fungal inhibition obtained from the dose–response curve. Then, 10 µL aliquots from those wells were transferred to potato dextrose agar (PDA) plates and incubated at 25 °C for 50 h with a 12 h photoperiod. After extracting the aliquots, 30 µL of resazurin was applied to each sample with visible growth to assess fungal viability, indicated by a color shift from purple to pink. The minimum fungicidal concentration (MFC) is defined as the lowest concentration at which no colony growth on the plate and no metabolic activity were detected. All analysis were realized in triplicate.

### 4.10. Microculture Method Based on Poisoned Media

The study employs an adapted version of a widely used microscopy technique to detect changes in fungal structures after exposure to plant and synthetic treatments via absorption. Two sterile PDA medium discs (6 mm × 6 mm) were positioned at each end of a sterile holder, and 30 µL of the following treatments were applied onto each disc: nanoemulsion of *Ocotea indecora* essential oil at 2500 µg/mL (C1-Ne-OiEO) and 312 µg/mL (C4-e-OiEO); *Ocotea indecora* essential oil (OiEO) at 10,000 µg/mL (C1-OiOE) and 1250 µg/mL (C4-OiOE); nanoemulsion blank (C-Ne, 2500 µg/mL); Tween 80 (C-EO, 242 µg/mL), and itraconazole (Itra, 10 µg/mL). After 10 min of applying the treatments, 10 µL of the spore solution from each strain, at a concentration of 7 × 10^6^ conidia/mL, was applied to the lateral surfaces of the treated and control discs (See Figure S6). Sterile coverslips were placed on each of the PDA discs to serve as a surface for the fungus to adhere to. 10 mL of sterile distilled water was pipetted over a layer of sterile gauze placed on the sides of each Petri plate to maintain humidity, which acts as an incubation chamber. 10 mL of sterile distilled water was pipetted over a layer of gauze. The Petri dishes were sealed and incubated at 25 °C for 96 h, with a 12 h photoperiod. Subsequently, 2 mL of formaldehyde was added to the gauze of each plate to limit fungal development and fix the structures on the microscope slides. The slides were observed under 10×, 20×, and 40× objectives using an Olympus BX43 optical microscope equipped with a QColor 3 camera (Olympus America Inc., Tokyo, Japan). Measurements of fungal structures in each micrograph were performed using ImageJ 1.52a software [58].

In addition to the descriptive microscopic analysis of *Th. ethacetica* based on Mbenoun et al. (2014), the following quantitative characteristics were evaluated: length and width of secondary conidia (*n* = 30), length and width of aleurioconidia (*n* = 20), and width of vegetative hyphae (*n* = 20) [52]. Since the conidia are ovoid in shape, the area (µm^2^) of secondary conidia and aleurioconidia was calculated using Equation (4).

(4) $$Ao\;({µm}^{2})=\pi *\frac{length\;(µm)}{2}*\;\frac{width\;(µm)}{2}$$

For compilation and statistical analysis, the data were exported to GraphPad Prism v.9.0. The normality of the data distribution was confirmed by the Kolmogorov–Smirnov test (*n* = 30) and the D’Agostino-Pearson test (*n* > 30) (*p* < 0.05). Parametric data were analyzed using ANOVA, while the Kruskal–Wallis test with post hoc analysis (*p* < 0.05) was used for non-parametric data. The experiment was performed as an independent duplicate.

## 5. Conclusions

This study evaluated the antibacterial and antifungal potential of *Ocotea indecora* essential oil and its nanoemulsion. The factorial design enabled the identification of optimal conditions for droplet formation, where low oil concentration, high surfactant content, and elevated sonication amplitude produced stable nanoemulsions. While nanoemulsification did not markedly enhance bacterial growth inhibition, it improved bactericidal activity against *S. aureus*. In contrast, a pronounced enhancement was observed against *Th. ethacetica*, where the nanoemulsion exhibited lower MIC values than the essential oil. Furthermore, both the oil and its nanoemulsion altered fungal morphology, impairing hyphal development and sporulation. Altogether, these findings highlight *O. indecora* derivatives as promising candidates for the development of novel antimicrobial agents, supporting their further investigation as natural alternatives for pharmaceutical and agricultural applications.

## Figures and Tables

**Table 1:** Chemical characterization of the *Ocotea indecora* essential oil from fresh leaves by GC-MS and GC-FID.

	RT (min)	AI_Rep_	AI_Lit_	Substances	Relative Abundance (%)
1	25.485	1265	1454	β-farnesene	2.95
2	29.309	1554	1549	Sesquirosefuran	91.61
3	30.094	1574	1570	Dendrolasin	0.84
4	30.219	1578	1577	Spathulenol	0.89
	TOTAL				96.29
	Sesquiterpene hydrocarbons				2.95
	Oxygenated sesquiterpene				93.34

RT, retention time; AI_rep_, arithmetic index reported; AI_Lit_, arithmetic index from Adams (2007) or El-sayed (2025).

**Table 2:** Average droplet size (nm) and polydispersity index from *O. indecora* formulations.

	Independent Variables			Dependent Variables	
Formulation	A	B	C	Droplet Size (nm)	Polydispersity Index
1	2.0	10.0	20.0	178.0 ± 2.8	0.107 ± 0.015
2	10.0	10.0	20.0	198.5 ± 3.0	0.121 ± 0.018
3	2.0	15.0	20.0	79.1 ± 11.8	0.045 ± 0.020
4	10.0	15.0	20.0	157.5 ± 2.2	0.248 ± 0.017
5	2.0	10.0	100.0	109.8 ± 5.8	0.205 ± 0.033
6	10.0	10.0	100.0	151.0 ± 3.6	0.287 ± 0.026
7	2.0	15.0	100.0	72.0 ± 10.4	0.029 ± 0.021
8	10.0	15.0	100.0	136.3 ± 2.0	0.208 ± 0.012
9	6.0	12.5	60.0	108.6 ± 6.1	0.122 ± 0.020
10	6.0	12.5	60.0	90.55 ± 9.4	0.081 ± 0.010
11	6.0	12.5	60.0	94.45 ± 7.0	0.077 ± 0.012

A, essential oil (%, *w*/*w*); B, Pluronic-L64 (%, *w*/*w*); C, amplitude.

**Table 3:** Stability of the *Ocotea indecora* nanoemulsion (Ne-OiOE) stored over 90 days at room temperature (25 °C), under refrigeration (8 °C), and in the climatic chamber (40 °C).

Days	25 °C ± 2		8 °C ± 2		40 °C ± 2	
	Size (nm)	PdI	Size (nm)	PdI	Size (nm)	PdI
0	76.56 ± 11.76	0.015 ± 0.010	78.36 ± 7.99	0.020 ± 0.005	81.05 ± 10.53	0.042 ± 0.008
7	83.46 ± 08.79	0.032 ± 0.011 ^b^	85.20 ± 8.86	0.055 ± 0.019	131.3 ± 5.76 ^e^	0.145 ± 0.023 ^f^
14	80.15 ± 10.50	0.054 ± 0.017	79.11 ± 12.30	0.107 ± 0.013 ^d^	96.83 ± 6.54	0.119 ± 0.010 ^f^
21	73.06 ± 09.82	0.051 ± 0.014	79.20 ± 10.10	0.023 ± 0.004	131.0 ± 6.01 ^e^	0.154 ± 0.025 ^f^
30	72.36 ± 09.88	0.056 ± 0.020	73.11 ± 10.28	0.034 ± 0.009	199.6 ± 1.81 ^e^	0.230 ± 0.047 ^f^
60	104.00 ± 04.63 ^a^	0.051 ± 0.013	94.84 ± 11.57	0.048 ± 0.014	128.8 ± 5.30 ^e^	0.161 ± 0.021 ^f^
90	71.81 ± 12.26	0.058 ± 0.012	122.1 ± 13.11 ^c^	0.210 ± 0.023 ^d^	135.9 ± 11.65 ^e^	0.146 ± 0.040 ^f^

^a^ (*p* 0.0149); ^b^ (*p* < 0.0003); ^c^ (*p* < 0.0001); ^d^ (*p* < 0.0001); ^e^ (*p* < 0.0001); ^f^ (*p* < 0.0001).

**Table 4:** Minimal inhibitory (MIC) and bactericidal (MBC) concentration (µg/mL) of the *Ocotea indecora* essential oil (OiEO) and nanoemulsion (Ne-OiOE) in bacterial strains.

Strains	OiEO		Ne-OiEO	
	MIC	MBC	MIC	MBC
*Staphylococcus aureus* ATCC 25923 (MSSA)	1024	2048	1024	1024
*Staphylococcus aureus* USA300 (MRSA)	2048	2048	2048	2048
*Staphylococcus epidermidis* ATCC 122282	2048	2048	048	2048
*Escherichia coli* ATCC 25922	>2048	>2048	>2048	>2048
*Enterobacter cloacae* ATCC 13047	>2048	>2048	>2048	>2048
*Pseudomonas aeruginosa* ATCC 27853	>2048	>2048	>2048	>2048
*Klebsiella pneumoniae* ATCC 13883	>2048	>2048	>2048	>2048

**Table 5:** Antifungal activity of nanoemulsion (Ne-OiEO) and essential oil of *O. indecora* (OiEO) on *Th. ethacetica*.

Treatment	Fungi	MIC (µg/mL) ^a^	IC_50_ (µg/mL) ^b^	95% IC_50_	R^2^	RMSE
Ne-OiEO	*Th. ethacetica*	625	322.7	286.5–363.4	0.90	12.18
OiEO	*Th. ethacetica*	2500	1252.0	1154–1355	0.98	5.65
Itraconazole *	*Th. ethacetica*	0.31	0.1682	0.1532–0.1890	0.92	10.4

^a^ MIC: Minimum Inhibitory Concentration; MIC values were determined by two criteria: concentration of treatment where no apparent growth was observed (qualitative criterion) and concentration of the treatment where the percentage of fungal inhibition was above 80% (quantitative criterion). ^b^ IC_50_: Concentration of treatment (µg/mL) that causes 50% inhibition of fungal growth; 95% IC_50_: 95% confidence intervals, the values are considered significantly different when the 95% CI fail to overlap; IC_50_ values and 95% confidence intervals obtained by non-linear regression from dose–response curves from three independent experiments; R^2^: coefficient of determination; RMSE, root mean square error. * Itraconazole was used as a positive inhibition control.

## Data Availability

The original contributions presented in this study are included in the article/Supplementary Materials. Further inquiries can be directed to the corresponding author.

## Supplementary Materials

The following supporting information can be downloaded at: https://www.mdpi.com/article/10.3390/ph18121909/s1, Figure S1. Total ion chromatogram of the essential oil of *Ocotea indecora* from leaves by GC-FID; Figure S2. Size distribution by intensity of the *O. indecora nanoemulsion* (Ne-OiOE) stored at (A) room temperature (25 °C), (B) under refrigeration (8 °C), and (C) climatic chamber (40 °C) over 90 days of preparation; Figure S3. Evaluating the stability of treatments by absorbance measurement in 96-well microplates at 25 °C; Figure S4. Microplates and replicate plates of *Thielaviopsis ethacetica* during the determination of the fungistatic and fungicidal activity of treatments; Figure S5. (A) Schematic of the 96-well microplate used in the microdilution assay; Figure S6. Schematic of the incubation plate used for the microculture method on the poisoned medium; Table S1. Analysis of variance (ANOVA) for the effects of oil concentration (A), Pluronic-L64 concentration (B), and sonication amplitude (C), as well as their interactions, on droplet size and polydispersity index (PdI) of *O. indecora* nanoemulsions; Table S2. Parameters of the dose-response curve from nanoemulsion of *Ocotea indecora* essential oil (Ne-OiEO) against *Thielaviopsis ethacetica*; Table S3. Parameters of dose-response curve from *Ocotea indecora* essential oil (OiEO) against *Thielaviopsis ethacetica*; Table S4. Parameters of the dose-response curve from itraconazole (Itra) against phytopathogenic fungi; Table S5. Experimental design for preparation of *O. indecora* formulations with Pluronic-L64; Chart S1. Statistical analysis of the effect of treatments on the secondary conidia (sC) area of *Thielaviopsis ethacetica*; Chart S2. Statistical analysis of the effect of treatments on vegetative hyphae width of *Thielaviopsis ethacetica*; Chart S3. Statistical analysis of the effect of treatments on aleurioconidia (aC) area of *Thielaviopsis ethacetica*.
