# Supplementary material for: Antibacterial and Antifungal Properties of Ocotea indecora Essential Oil and Its Nanoemulsion
Source: Pharmaceuticals (Basel). 2025 Dec 18;18(12):1909. doi: 10.3390/ph18121909 (PMC12736034; doi:10.3390/ph18121909)
Supplement: Supplementary file 1 [file pharmaceuticals-18-01909-s001.zip › pharmaceuticals-3948315-supplementary.pdf]

pharmaceuticals

Research article

Antibacterial and Antifungal Properties of *Ocotea indecora* Essential Oil and its Nanoemulsion

Francisco Paiva Machado [1], Julia C. Scaffo [1,2], Leonardo A. Pinto [1], Renata F. A. Pereira [2], Sorele Fiaux [3], Luiz Antonio M. Keller [4], Eduardo Ricci-Júnior [5], Ana Paula dos Santos Matos [5,6], Fabio Aguiar-Alves [2,7], Caio P. Fernandes [8,*], Jorge A. D. Duarte [3,9] and Leandro Rocha [1]

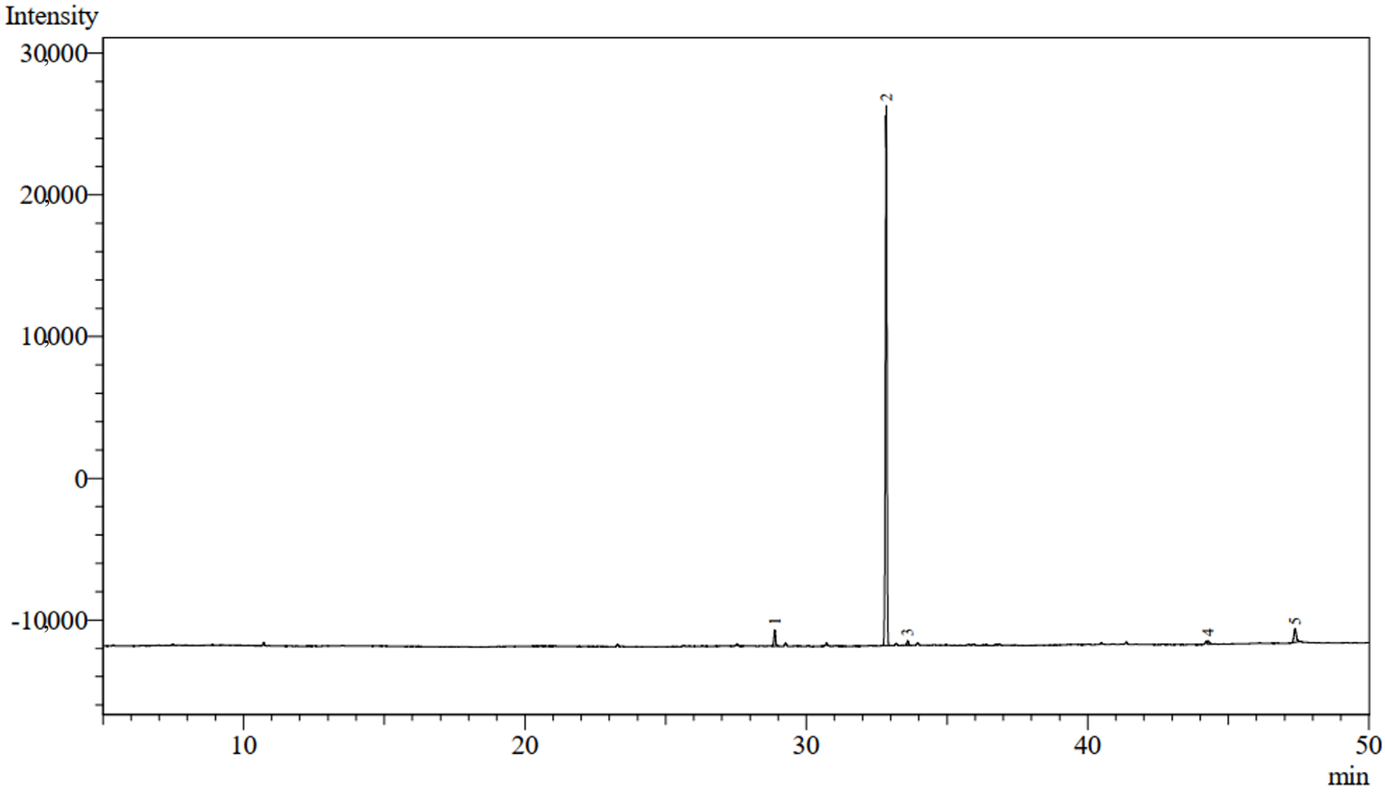

Figure S1. Total ion chromatogram of the essential oil of *Ocotea indecora* from leaves by GC-FID; 1 β-farnesene; 2 Sesquirosefuran; 3 Dendrolasin; 4 Spathulenol; 5 non-identified.

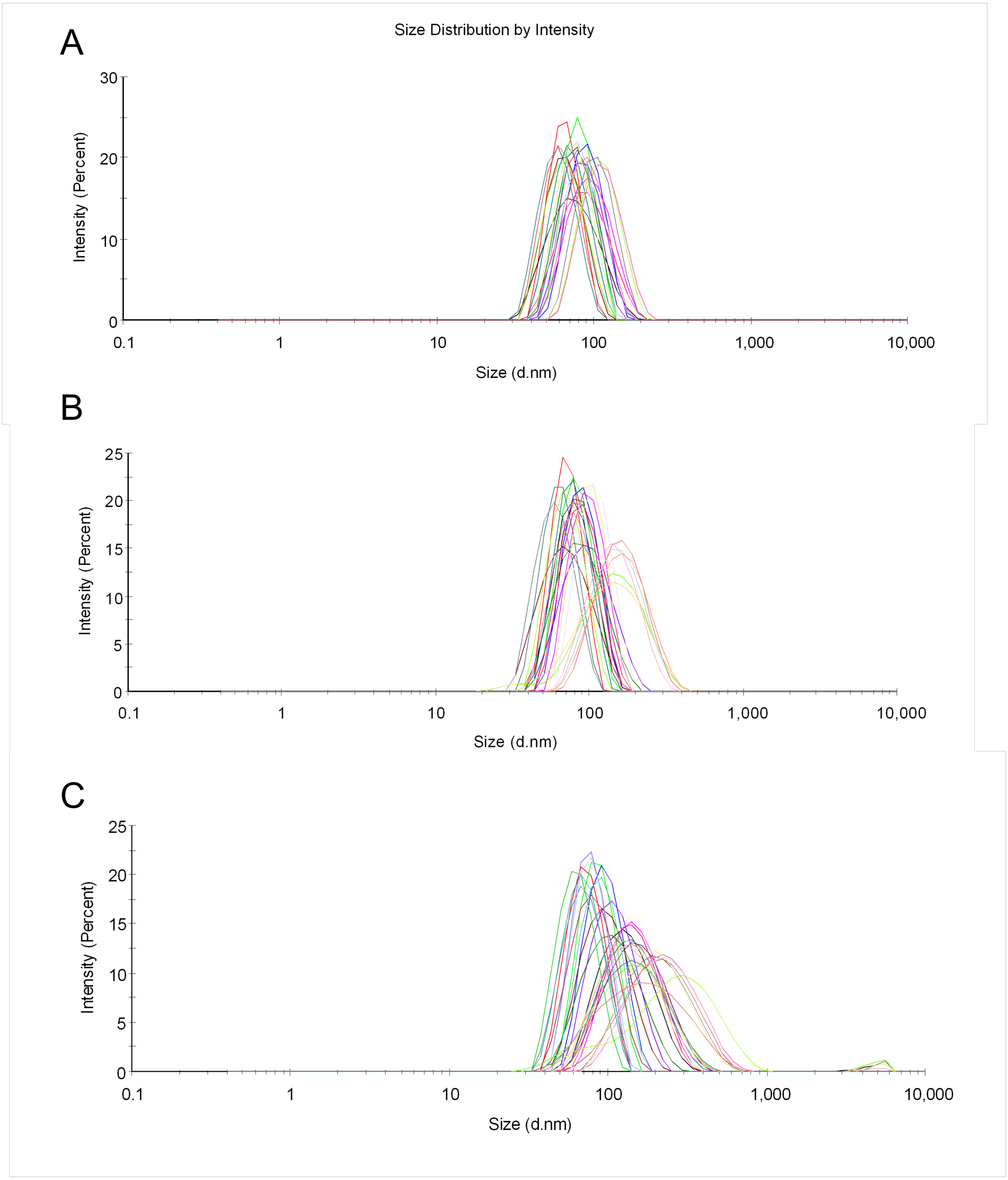

Figure S2. Size distribution by intensity of the *O. indecora* nanoemulsion (Ne-OiOE) stored at (A) room temperature (25 °C), (B) under refrigeration (08 °C), and (C) climatic chamber (40 °C) over 90 days of preparation.

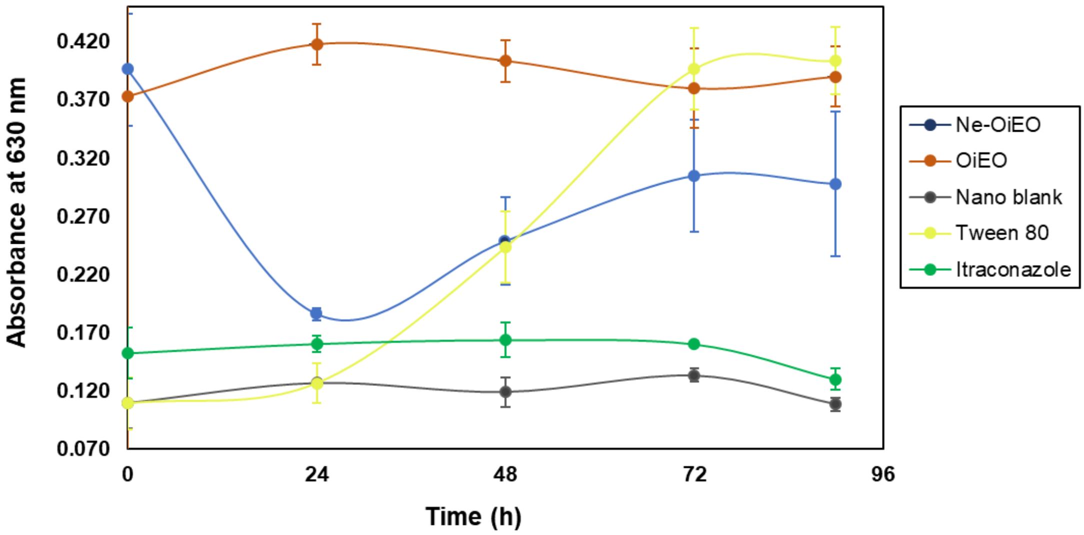

Figure S3. Evaluating the stability of treatments by absorbance measurement in 96-well microplates at 25 °C.

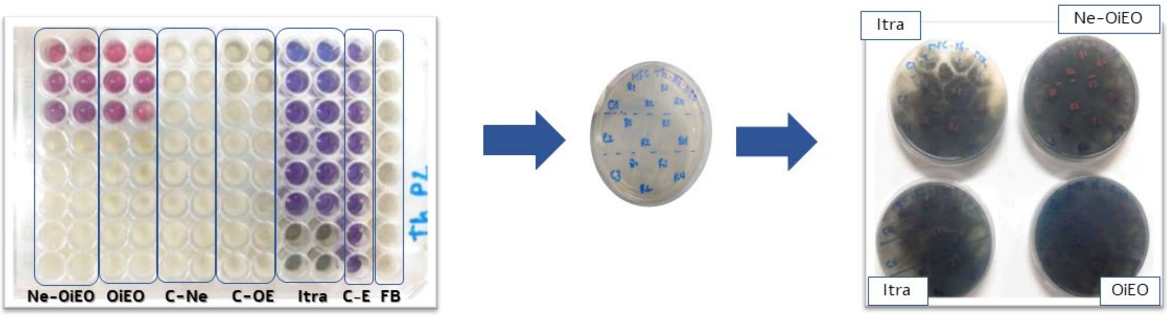

Figure S4. Microplates and replicate plates of *Thielaviopsis ethacetica* during the determination of the fungistatic and fungicidal activity of treatments. Treatments: *Ocotea indecora* essential oil nanoemulsion (Ne-OiEO), nanoemulsion blank (C-Ne), *Ocotea indecora* essential oil (OiEO), Tween 80 (C-EO), Itraconazole (Itra), sterility control (C-E), and fungal blank (FB). The microplates from the microdilution experiment are shown in Figure 4S. Micro wells with no visible resazurin were used to assess the cellular activity of the pathogens. Fungal development is indicated by wells that are white, yellow, or grey in coloration. When compared to sterile wells, pink wells indicate the existence of spore metabolic activity, while purple wells show the absence of cellular activity. Aliquots from the wells with no visible growth were duplicated on sterile nutrient medium plates and incubated at 25 °C for 48 h. After replating, fungal growth was observed on the plates, indicating that the treatments had no effect on fungal activity.

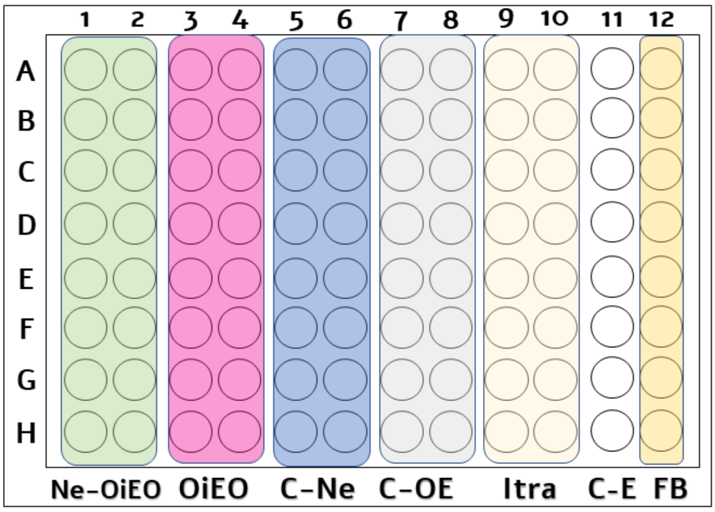

Figure S5. (A) Schematic of the 96-well microplate used in the microdilution assay. Treatments: *Ocotea indecora* essential oil nanoemulsion (Ne-OiEO), nanoemulsion blank (C-Ne), *Ocotea indecora* essential oil (OiEO), Tween 80 (C-EO), Itraconazole (Itra), sterility control (C-E), and fungal blank (FB).

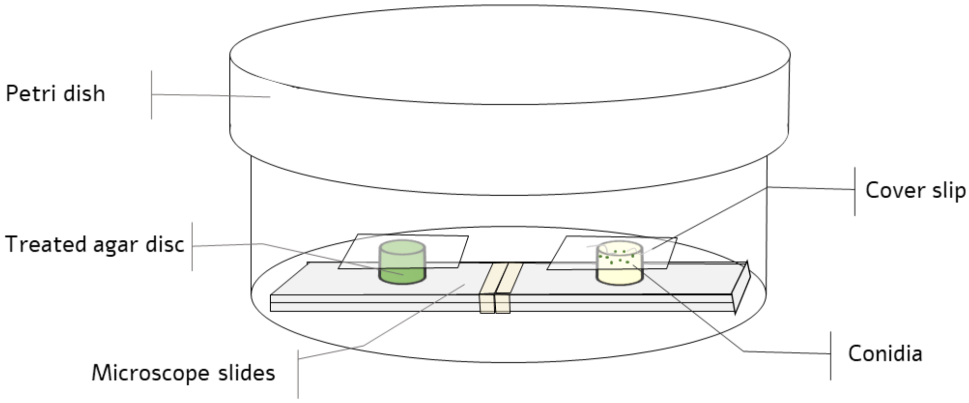

Figure S6. Schematic of the incubation plate used for the microculture method on the poisoned medium.

Table S1. Analysis of variance (ANOVA) for the effects of oil concentration.

	**Droplet size (nm)**				**Polydispersity index**			
	SS	MS	F	p-value	SS	MS	F	p-value
Curvature	3052.38	3052.384	33.878	**0.0283**	0.008637	0.008637	13.9227	0.0649
A	5224.46	5224.464	57.917	**0.0168**	0.028561	0.028561	46.0406	**0.0210**
B	4629.14	4629.144	51.318	**0.0190**	0.004513	0.004513	7.274	0.1144
C	2593.44	2593.440	28.751	**0.0331**	0.005408	0.005408	8.7188	0.0981
AB	820.94	820.935	9.101	0.0946	0.010225	0.010225	16.4828	0.0560
AC	5.51	5.511	0.061	0.8278	0.000242	0.000242	0.3901	0.5960
BC	953.97	953.971	10.575	0.0830	0.012800	0.012800	20.6341	**0.0452**
Lack of Fit	151.03	151.032	1.674	0.3249	0.001058	0.001058	1.7055	0.3216
Pure Error	180.41	90.206	-	-	0.001241	0.000620	-	-
Total SS	17611.29	-	-	-	0.072683	-	-	-

Significant values ($p < 0.05$) are highlighted in bold; A, Pluronic-L64 concentration; B, and sonication amplitude; C, as well as their interactions, on droplet size and polydispersity index (PdI) of *O. indecora* nanoemulsions.

Table S2. Parameters of the dose-response curve from nanoemulsion of *Ocotea indecora* essential oil (Ne-OiEO) against *Thielaviopsis ethacetica.*

**Log(inhibitor) vs. normalized response -- Variable slope**	***Thielaviopsis ethacetica***
Best-fit values	
LogIC50	2.509
HillSlope	4.710
IC50	322.7
95% CI (profile likelihood)	
LogIC50	2.457 to 2.560
HillSlope	2.571 to ???
IC50	286.5 to 363.4
Goodness of Fit	
Degrees of Freedom	30
R squared	0.9069
Sum of Squares	4600
Sy.x	12.38
RMSE	12.18
Number of points	
# of X values	32
# Y values analyzed	32

Table S3. Parameters of dose-response curve from *Ocotea indecora* essential oil (OiEO) against *Thielaviopsis ethacetica.*

Log(inhibitor) vs. normalized response -- Variable slope	*Thielaviopsis ethacetica*
Best-fit values	
$LogIC_{50}$	3.098
HillSlope	2.328
IC_{50}	1252
95% CI (profile likelihood)	
$LogIC_{50}$	3.062 to 3.132
HillSlope	1.963 to 2.821
IC50	1154 to 1355
Goodness of Fit	
Degrees of Freedom	30

R squared	0.9813
Sum of Squares	989.5
Sy.x	5.743
RMSE	5.650
Number of points	
# of X values	32
# Y values analyzed	32

Table S4. Parameters of the dose-response curve from itraconazole (Itra) against phytopathogenic fungi.

**log(inhibitor) vs. normalized response -- Variable slope**	***Thielaviopsis ethacetica***
Best-fit values	
$LogIC_{50}$	-0.7741
HillSlope	4.418
IC50	0.1682
95% CI (profile likelihood)	
$LogIC_{50}$	-0.8148 to -0.7236
HillSlope	2.893 to ???
IC_{50}	0.1532 to 0.1890
Goodness of Fit	
Degrees of Freedom	30
R squared	0.9227
Sum of Squares	3351
Sy.x	10.57
RMSE	10.40
Number of points	
# of X values	32
# Y values analyzed	32

Table S5. Experimental design for preparation of *O. indecora* formulations with Pluronic-L64.

**Factor**	**Level**		
	**Low (-1)**	**Medium (0)**	**High (+1)**
Independent variables			
(A) Essential oil (%)	2.0	6.0	10.0
(B) Pluronic L-64 (%)	10.0	12.5	15.0
(C) Amplitude (%)	20.0	60.0	100.0
Dependent variables			
Droplet size (nm)			
Polydispersity index			

Chart S1. Statistical analysis of the effect of treatments on the secondary conidia (sC) area of *Thielaviopsis ethacetica*.

**Graphical result of the normal distribution test**

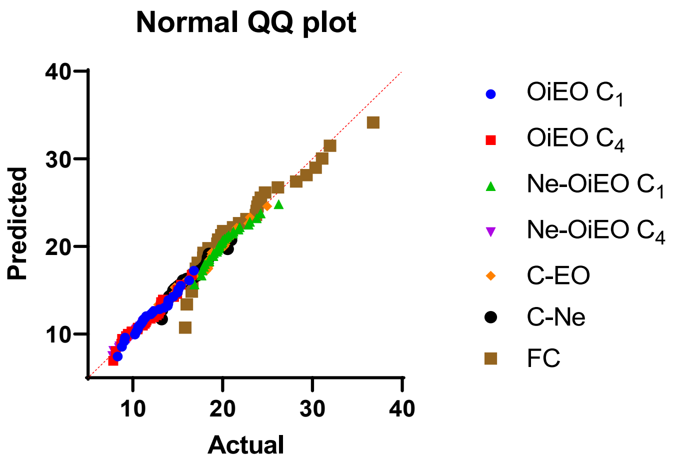

Variance analysis result for parametric data

ANOVA table	SS	DF	MS	F (DFn, DFd)	P value
**Treatment (between columns)**	4030	6	671.7	F (6, 203) = 80.40	P<0.0001
**Residual (within columns)**	1696	203	8.354		
**Total**	5726	209			
**Data summary**					
Number of treatments (columns)	7				
Number of values (total)	210				

Post hoc analysis

Number of families	1
Number of comparisons per family	6
Alpha	0.05

Dunnett's multiple comparisons test	Mean Diff.	95.00% CI of diff.	Below threshold?	Summary	Adjusted P Value
FC vs. OiEO C_1	10.10	8.169 to 12.03	Yes	****	<0.0001
FC vs. OiEO C_4	10.60	8.663 to 12.53	Yes	****	<0.0001
FC vs. Ne-OiEO C_1	2.199	0.2667 to 4.131	Yes	*	0.0186
FC vs. Ne-OiEO C_4	12.38	10.45 to 14.31	Yes	****	<0.0001
FC vs. C-EO	3.459	1.527 to 5.391	Yes	****	<0.0001
FC vs. C-Ne	6.222	4.290 to 8.154	Yes	****	<0.0001

Chart S2. Statistical analysis of the effect of treatments on vegetative hyphae width of *Thielaviopsis ethacetica*.

Graphical result of the normal distribution test

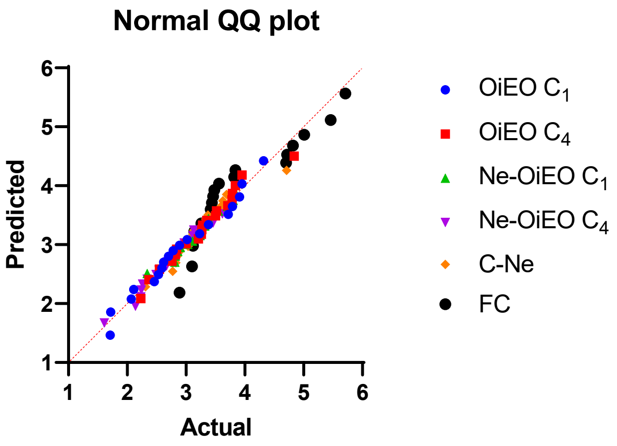

**Variance analysis result for parametric data**	
**Kruskal-Wallis test**	
P value	0.0001
Exact or approximate P value?	Approximate
P-value summary	***
Do the medians vary significantly? (P < 0.05)?	Yes
Number of groups	6
Kruskal-Wallis statistic	25.09
Data summary	
Number of treatments (columns)	6
Number of values (total)	120

Post hoc analysis

Dunn's multiple comparisons test

Multiple comparisons	Mean rank diff.	Significant?	Summary	Adjusted P Value
FC vs. OiEO C_1	38.13	Yes	**	0.0026
FC vs. OiEO C_4	20.88	No	ns	0.2886
FC vs. Ne-OiEO C_1	23.33	No	ns	0.1698
FC vs. Ne-OiEO C_4	51.48	Yes	****	<0.0001
FC vs. C-Ne	22.50	No	ns	0.2040

Chart S3. Statistical analysis of the effect of treatments on aleurioconidia (aC) area of *Thielaviopsis ethacetica.*

Graphical result of the normal distribution test

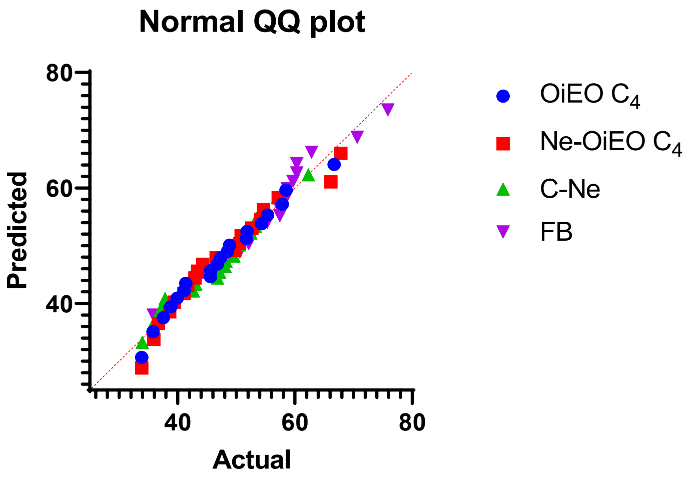

Variance analysis result for parametric data

ANOVA table	SS	DF	MS	F (DFn, DFd)	P value
**Treatment (between columns)**	1017	3	338.9	F (3, 76) = 4.528	P=0.0056
**Residual (within columns)**	5688	76	74.85		
**Total**	6705	79			

Post hoc analysis

**Number of families**	**1**
Number of comparisons per family	3
Alpha	0.05

Dunnett's multiple comparisons test	Mean Diff.	95.00% CI of diff.	Below threshold?	Summary	Adjusted P Value
FB vs. OiEO C_4	8.372	1.814 to 14.93	Yes	**	0.0086
FB vs. Ne-OiEO C_4	8.330	1.772 to 14.89	Yes	**	0.0089
FB vs. C-Ne	7.976	1.418 to 14.53	Yes	*	0.0129
